# At-risk advertising by Australian chiropractors and physiotherapists

**DOI:** 10.1186/s12998-019-0247-x

**Published:** 2019-06-13

**Authors:** J. Keith Simpson

**Affiliations:** Murdoch University, School of Chiropractic, South Street, Murdoch, 6150 Australia

**Keywords:** Chiropractor, Physiotherapist, Advertising breaches, Misleading and deceptive conduct

## Abstract

**Background:**

Society expects professionals to promote their businesses in an ethical manner, refraining from misleading or deceptive marketing due to the potential to harm members of the community. In Australia this expectation resides in the Australian registration board advertising guidelines or the Health Practitioner Regulation National Law. Registration board data indicate there are many health care professionals failing to meet these expectations. The aims of this research were to determine the frequency, type and nature of at-risk advertising by Australian chiropractors and physiotherapists and whether there is a correlation between professional association membership and advertising guideline compliance.

**Method:**

A cross sectional audit examining practitioner advertising was performed on representative samples of Australian chiropractors and physiotherapists. Two auditors examined advertising by 380 physiotherapists and 359 chiropractors for material potentially in breach of the regulatory authorities’ advertising guidelines. The advertising appeared on practitioner websites and linked Facebook pages.

**Results:**

Two-hundred and fifty-eight (72%) audited chiropractors and 231 (61%) audited physiotherapists had breaches of the Advertising Guidelines on their websites and linked Facebook pages. The frequency of breaches by chiropractors was higher. The type and nature of the breaches by chiropractors was potentially more harmful. Membership in a professional association influenced neither the frequency nor the severity of breaches with chiropractors.

**Discussion:**

Advertising breaches were common in both samples even though regulators and professional associations provide practitioners with explicit information on how to comply with advertising guidelines. Breaches by chiropractors were more numerous and more serious due to their greater potential to lead consumers to make inappropriate and potentially harmful healthcare decisions.

Stronger enforcement strategies may have a positive effect on compliance.

## Background

Advertising by health professionals is an integral part of practise. Providing consumers with ethical accurate advertising assists with making informed health related decisions.

Chiropractors and physiotherapists are amongst Australia’s 15 regulated health professions. All must abide by the same advertising guidelines. The guidelines stipulate what constitutes unacceptable advertising. Unacceptable advertising includes advertising that is false, misleading or deceptive or likely to deceive. Unacceptable advertising has the potential to cause harm. Recent Australian court cases have highlighted the potential harm to consumers when health care providers publish false advertising [1, 2]. Although the regulatory authorities’ annual reports provide the numbers of advertising complaints made for each profession, they contain no details about the frequency, type and nature of the complaints.

This research examined advertising by a representative sample of Australian chiropractors and physiotherapists. Practitioner websites and linked Facebook pages were audited. It reports on the frequency, type and nature of advertising at risk of being non-compliant with the advertising guidelines. In addition, this research determined whether there is a correlation between professional association membership and advertising guideline compliance.

### Advertising and the National law in Australia

Since 2010 all registered Australian health care providers have had uniform nationwide legislation – The National Law (NL). The NL outlines the regulatory obligations for advertising a regulated health service. Obligations include: advertising must not be false, misleading or deceptive or likely to be misleading or deceptive and must not include testimonials; any claims made must be able to be substantiated; offers of gifts, discounts or other inducements must come with terms; advertising must neither create and unreasonable expectation of beneficial treatment nor directly or indirectly encourage the indiscriminate or unnecessary use of regulated health services [3].

Under the NL, the definition of a regulated health service is very broad and is not restricted to direct clinical services. Uniform Advertising Guidelines (AG) are in place for each of the 15 regulated health professions governed by its own Board. A breach of advertising provisions of the NL is a criminal offence punishable by fine. Other enforcement approaches include the respective Board placing restrictions on an individual’s registration and their ability to practise. Legislative authorities in other countries including the United States of America, Canada, and the United Kingdom have also legalised advertising by health care professionals and have similar regulations governing professionals’ advertising.

### Understanding misleading or deceptive or likely to be misleading or deceptive

Before considering the regulatory authorities’ compliance and enforcement strategy, it is appropriate to consider one of the most challenging aspects of the AG, namely what is meant by misleading or deceptive. The Chiropractic Board of Australia (CBA) advises that misleading someone may include lying to them, leading them to a wrong conclusion, creating a false impression, leaving out important information, or making false or inaccurate claims [4]. And the CBA correctly points out, “the ways which advertising can be false, misleading or deceptive are almost limitless” [4]. Additionally, silence may constitute misleading or deceptive conduct where there is a duty to reveal relevant facts [5]. The courts have determined that people who are misled are almost by definition deceived as well. Regarding the phrase ‘likely to mislead’: there is no requirement to prove that a person was misled or deceived, rather, the sufficient test is whether there is a real and not remote chance to mislead. As far as who is misled or deceived, the courts have determined that the misleading and deceptive conduct provisions are concerned with the public at large, or as it is sometimes referred to, the “target audience”. Members of the “target audience” include:

the astute and the gullible, the intelligent and the not so intelligent, the well-educated as well as the poorly educated, men and women of various ages pursuing a variety of vocations. … all persons exposed to the conduct should be considered although conduct which is only likely to mislead or deceive an extraordinarily stupid person would not fall within the ambit of the provisions [6].

The courts will consider whether a reasonably significant number of potential purchasers would be likely to be misled or deceived. Intent is not a necessary element for conduct to breach the misleading conduct provisions. Conduct may be regarded as misleading or deceptive even if the originator of the conduct did not intend to mislead or deceive members of the target audience. The test is objective. The factor is conduct taken at face value [7].

### AHPRA’s compliance and enforcement strategy: education & engagement

The Australian Health Practitioner Regulation Agency (AHPRA) prosecutes breaches of the AG. The high volume of complaints received by AHPRA during 2016 triggered the establishment of a dedicated Advertising Compliance Team which works closely with the Legal Team and the Policy and Communications Team. To assist in their decision making, AHPRA utilise experts to evaluate advertising claims.

AHPRA considers that education and engagement are effective tools as part of its strategy to achieve behaviour change and compliance with the regulations. AHPRA has developed advertising education tools accessible via the AHPRA website under the heading: Check, Correct, Comply. This section of the website includes numerous examples of non-compliant advertising common to all professions and examples specific to chiropractors. In addition, the National Boards consider that some words have a greater capacity to mislead or deceive when used in advertising and recommend that advertisers be cautious when using them. The “words to be wary [of]: cure, safe, effective, and can help/ improve/treat or effectively treats” [8]. In addition, the Chiropractic Board of Australia has issued position statements on Paediatric Care [9] and on Care of the Pregnant Patient [10] which deal with inappropriate claims of benefit and antivaccination advice.

In response to a recognised need to reduce non-compliant advertising AHPRA announced a pilot audit of chiropractic and dentist advertising commencing in the 2019 registration period. This step marks a shift from reactive enforcement to proactive enforcement and is expected to improve compliance across the entire sector. The data generated by AHPRA will inform a review of the compliance strategy, identify profession-specific differences in compliance rates, inform future strategic directions and ensure sustainable change [11].

### Conduct notifications & advertising breaches: the Australian scene

In Australia there are 657,621 registered health practitioners across 15 professions. The 5167 registered chiropractors make up 0.8% of the total health practitioner registrant base, while the 28,885 registered physiotherapists make up 4.5% of the base [12].

Advertising complaints are considered separately from conduct complaints. AHPRA Annual Reports covering the period 2013–2017 [12–15] demonstrate the growing challenge of regulatory control of practitioner advertising. (Table 1).

**Table 1:** Advertising Complaints by Profession: 2013–2017

Advertising Complaints	2013/14	2014/15	2015/16	2016/17	2017/18
Total	547	300	1013	1895	1043
Chiropractic	186	120	601^a^	162	15
Physiotherapy	28	25	44	903^b^	8

Explanatory Notes:

^a^No explanation could be found for the spike in advertising complaints against chiropractors

^b^ According to the Physiotherapy Board of Australia (PBA) the 1300% increase in advertising complaints over the previous year was due to the lodgement of bulk complaints by several organisations about suspected advertising breaches [49]. The PBA further stated that the vast majority of the 903 advertising complaints did not require action [50]

A clear picture exists regarding conduct complaints against chiropractors and physiotherapists in Australia [16] and where the complaints come from [17] providing regulatory bodies with valuable information for developing preventive strategies. Recent research demonstrates that the Australian chiropractic profession generates a disproportionate number of professional conduct complaints. Professional conduct refers to: procedures, treatment, communication, assessment, diagnosis and other professional conduct issues (advertising and titles), sexual boundaries, honesty in fees, interpersonal behaviour, records and reports. Chiropractors have a higher rate of conduct complaints than psychologists, optometrists, podiatrists, nurses, physiotherapists or occupational therapists per 100 practitioners [18]. Only dentists and medical practitioners generate more complaints per 100 practitioners [18]. The chiropractic conduct complaints are 6 times higher than those of physiotherapists and 3 times higher than those of osteopaths [16].

The reactive nature of AHPRA’s approach has obvious limitations. Spittal, Bismark and Studdert [19] suggest that a predictive proactive approach to identify practitioners at risk of becoming the subject of repeated patient complaints would assist medicolegal agencies such as malpractice insurers, medical boards and complaints handling bodies in fulfilling their role of protecting the public. Spittal et al. have developed an algorithm for predicting a doctor’s risk of conduct complaints. Dubbed the PRONE (Predicted Risk of New Event) score, their algorithm may be adaptable to other health care professions such as chiropractors and physiotherapists [19].

A less clear picture exists regarding advertising complaints. Regulatory authority reports confirm that Australian chiropractors and physiotherapists are the subject of significant numbers of advertising complaints [12–15]. In 2016 these matters were noticed by the Australian Health Minister’s Advisory Council which issued a ‘please explain’ notice to the Chiropractic Board of Australia (CBA) [20]. In response, the CBA acknowledged the unacceptable false advertising practices of some chiropractors, stating:

There is no evidence chiropractic care benefits babies or can treat them for medical conditions and there is not enough evidence to suggest it [chiropractic] can achieve general wellness or treat various organic diseases and infections.

it [CBA] was concerned about a number of practitioners who were falsely advertising that chiropractic care for spinal problems could also treat a range of other ailments [21].

Emphasising the need for concern, a 2018 high profile Australian case establishes that serious breaches do occur and that misleading advertising by health practitioners can harm members of the community [2]. Until now no research has been conducted into the frequency, type and nature of advertising breaches by chiropractors or physiotherapists in Australia.

## Objectives

This research had two objectives: 1) to determine the frequency, type and nature of Australian advertising guideline breaches by Australian chiropractors and physiotherapists and 2) to determine if there is a correlation between compliance with advertising guidelines and professional association membership.

## Methods

This study was a cross-sectional audit of chiropractors’ and physiotherapists’ online marketing material examining practitioner compliance with advertising guidelines. The audit was conducted over a 4-week period between July 15, 2018 and August 15, 2018.

In Australia, chiropractors and physiotherapists are registered by separate regulatory boards – CBA and PBA – under the umbrella of a National Health Practitioner Regulation Scheme. There were 5284 registered chiropractors and 31,995 registered physiotherapists across Australia as at 30 June 2018. Sample size was calculated using Table 1 in Krejcie and Morgan [22] and confirmed using the National Statistical Service online calculator using a 95% confidence level and 5% confidence interval [23]. For the chiropractic population of 5284 a sample size of 359 was required while for the physiotherapist population of 31,995 a sample size of 380 was required.

Advertising appearing on the websites and associated Facebook pages of 359 chiropractors and 380 physiotherapists was audited. Because of the prevalence of group private practice (in 2016, 54.1% of chiropractors and 28.1% of physiotherapists were in group private practice [24, 25]) 151 chiropractor websites and 72 physiotherapist websites and linked Facebook pages were inspected to obtain data on 359 chiropractors and 380 physiotherapists.

### Data collection

Two auditors collected the data. Both auditors have been registered chiropractors for over 30 years and have extensive experience in professional regulation compliance and enforcement matters. Each auditor collected data for ½ of each sample (≈179 chiropractors, 190 physiotherapists) with minimal overlap. When overlap occurred, there was agreement on numbers and categories of breaches. If a question arose regarding how to classify a particular aspect of a practitioner’s advertising, the auditors discussed the matter and reached a consensus. These points were a guide:

- Where breaches in multiple categories were found only one example per practitioner per category was recorded.
- Where multiple breaches were found in a single category, only one example was recorded.
- The auditors were careful to attribute breaches to individual practitioners where it was clear the utterance applied to an individual, and to attribute the breach to all practitioners where the utterance reasonably applied to all practitioners in the practice.
  ◦ Example: Use of association membership as postnominals or a specialisation claim was attributed only to the individual practitioner.
  ◦ Example: A claim made on a home page or FAQ section was attributed to all practitioners in the practice.
- If a claim could be allocated into more than one category, it was registered in the category deemed to have the greatest potential for harm. For example, the claim:
  If you’re a frequent flyer, make the best of it and remember to come in for your chiropractic adjustments often.
  is a misleading unsubstantiated claim breach and a breach of the “encourages inappropriate, indiscriminate, unnecessary or excessive” use category. The greatest potential for harm would be by encouraging inappropriate or excessive use of a health service therefore this example was only counted as a breach of that category. (Please refer to Breach Categories below for details of how breaches were classified).

### Locating and auditing practitioner websites & linked facebook pages

A three-step process was followed to locate practitioner websites and linked Facebook pages.

Step 1.

Practitioner lists were created using the appropriate Board’s search engine: 359 chiropractors and 380 physiotherapists. The lists were compiled by entering the letter A in the Board’s “Check your health practitioner is registered” search field. This retrieved a list of all registrants with a surname beginning with A. Every 5th name was used to compile the list. The same procedure was used with each letter of the alphabet until the required number of names was retrieved.

Step 2.

The practitioner’s practice was located using a web search either for the practitioner by name or by suburb. If the web search for the practitioner by name located their practice website, the audit began. If the search failed to locate the practitioner’s website, a second web search using postcode, suburb name and practitioner name was conducted. Typically, these approaches located the practitioner’s website. In the limited number of cases where a practitioner’s website could not be located, the next name on the practitioner list was used. It is estimated that less than 10% of practitioners’ names resulted in a negative search.

Step 3.

The practitioner’s website and associated Facebook page were audited. Typically, practitioner websites included these sections, each of which was audited:

- Home page
- About/Meet the Team
- What is chiropractic/physiotherapy?
- Frequently Asked Questions
- Conditions treated
- Blog posts
- How it works/Research^1^
- Testimonials
- Facebook
  ◦ Videos
  ◦ Reviews
  ◦ Info & Ads
  ◦ Photos

### Association membership

Membership in a professional association was determined by using the “locate a chiropractor” search engine for both the Chiropractors Association of Australia and Chiropractic Australia. The “Find a Physio” search engine of the Australian Physiotherapy Association (APA) proved unreliable and the APA would not assist with membership details, so this aspect of the research was abandoned.^2^

### Breach categories

The Guidelines For Advertising Regulated Health Services (AG) [26] were jointly developed and are used by all National Boards under section 39 of The National Law. The guidelines were developed to help practitioners and others understand their obligations when advertising a regulated health service. Before March 2014 when the AG were revised, Section 5 of the 2014 AG (§5) was entitled “What is unacceptable advertising?” [3, 27]. This section described examples of unacceptable advertising providing practitioners with a clear indication of what the boards considered objectionable advertising practices. In other words, advertising at-risk of breaching the AG. For this research, §5 provided the criteria against which to audit practitioner advertising.

In addition, the AG contain statements on the substantiation of claims, specialization claims, advertising titles, qualifications or memberships, and using scientific information in advertising.

After considering the explanatory notes within the 2014 and subsequent AG, 32 categories of unacceptable advertising emerged. To assist with coding breaches, the §5 category “Mislead, either directly, or by implication, use of emphasis, comparison, contrast or omission” was subdivided into 9 classes (c1-c9) and classified as Major or Minor based on information provided within the AG and explanatory notes. (Table 2).

**Table 2:** Misleading Claims: Major or Minor Misleading Classes

Category	Minor: Unlikely to harm	Major: Likely to harm
c1 Association membership presented as postnominals.	Persons displaying association membership in this way are presumably abiding by their association’s code of ethics when dealing with clients so the potential for harm is lessened.	
c2 Use of the title Dr. without professional clarification.	Relatively unlikely that a member of the public would be misled into thinking a chiropractor using the title Dr. is also a medical practitioner.	
c3 Use of Doctor of Chiropractic or DC without holding the qualification but having graduated with a chiropractic qualification from an accredited chiropractic program.	Unlikely to mislead the target audience because members of the public would be unlikely to know the distinction although if they misrepresent their academic qualifications they may do so in other areas.	
c4 Specialisation claim.	Practitioners using this designation presumably have a special interest in a particular area however this does not necessarily mean qualifications that would deem them ‘specialists’ and hence the public may be misled.	
c5 Claims to affect positioning of an unborn child.^a^		Any advertisement claiming or implying that a technique can affect an obstetric breech presentation is misleading and potentially harmful.
c6 Misuse of the literature.		High likelihood of misleading the target audience because almost inevitably the advertiser omits critical information from the literature cited or fails to provide a balanced report of the literature.
c7 Failure to mention possible adverse outcomes.		Failure to mention possible adverse outcomes has a relatively high chance of misleading the target audience into believing that a form of treatment is free from possible adverse outcomes.
c8 Making unsubstantiated claims.		An advertiser must have reasonable grounds for making a claim of effectiveness.^b^
c9 Misrepresenting awards. *Eg.* Presenting a business award as though it is a clinical award.	A practice which has won a business award may be more likely to comply with required practice standards and is therefore less likely to mislead patients in clinical practice areas.	

Explanatory Note:

^a^The CBA published clear advice on advertising care of pregnant patients in its March 2016 statement on advertising:

Chiropractors are not trained to apply any direct treatment to an unborn child and should not deliver any treatment to the unborn child. Chiropractic care must not be represented or provided as treatment to the unborn child as an obstetric breech correction technique [51].

^b^The courts have shown that determining what constitutes reasonable grounds is not left to the discretion of the advertiser. Rather, reasonable grounds in the view of the courts equates to “sufficient scientific knowledge” [52].

### Recording and data analysis

The raw data was recorded onto separate Excel spreadsheets for each profession. Data captured included: practice URL; practice location by State; practitioner name(s); and breaches. NVivo 12, a qualitative data analysis software package, was used to organise and analyse the breaches data for both professions.

Breaches were identified by the corresponding letter or number from the 2014 AG §5 + Table 2. When a contravening statement was located it was copied and pasted into the Excel spreadsheet. The following example was recorded as a breach of ‘b’ – “encourage (directly or indirectly) inappropriate, indiscriminate, unnecessary or excessive use of health services” by a chiropractor.

If you’re a frequent flyer, make the best of it and remember to come in for your chiropractic adjustments often.

## Results

No practitioners from either profession emerged from the Northern Territory during the sampling process. Practitioner webpages were audited from all other Australian jurisdictions.

### Breaches by the numbers

**Fig. 1:**
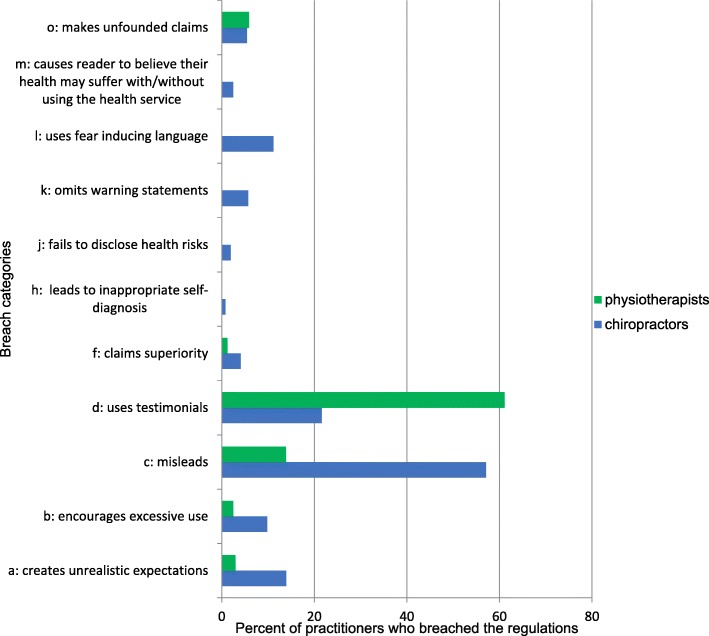
Types of Breaches by Category of Practitioner

**Fig. 2:**
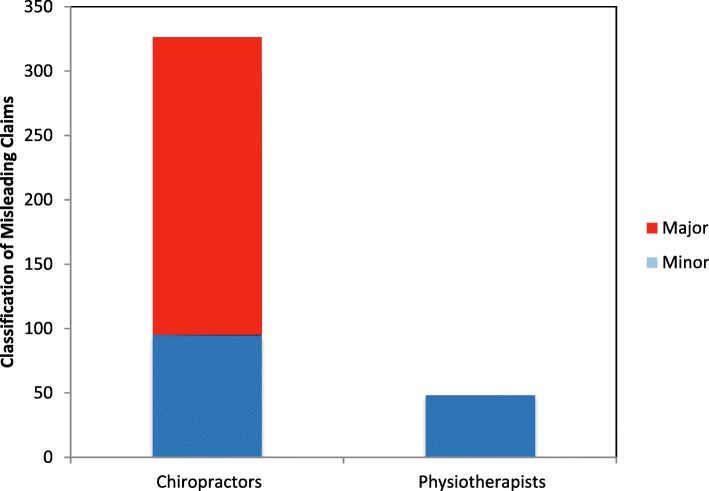
Number of Minor and Major misleading claims by category of practitioner

Seventy-two percent (259) of audited chiropractors and 61% (232) of audited physiotherapists had breaches in one or more categories. Chiropractors had breaches in 11 of the 15 categories with the most frequent being misleading representations such as unsubstantiated claims and misuse of the scientific literature. Physiotherapists had breaches in 6 of the 15 categories with the most frequent being testimonials and misleading representations such as displaying association membership as postnominals or specialisation claims. There were no breaches in categories e, g, i, and n by either category of practitioner (Fig. 1). Two hundred and five chiropractors (57%) made misleading claims on their website or linked Facebook page and 78 physiotherapists (20%) did so. Of the 326 misleading claims made by chiropractors 231 (71%) were considered major misleading claims based on the criteria outlined in Methods. Physiotherapists made no major misleading claims (Fig. 2). The results from each §5 breach category with de-identified examples from practitioner websites and Facebook pages appear in Tables 3, 4, 5, and 6.

**Table 3:** All Breaches by Chiropractors (Number and %) with Examples

Section 5 of the 2014 AG (§5) Category	Chiropractors with Breaches: Number & (%)	Example
(a) Create unwarranted and unrealistic expectations about service effectiveness	75 (21%)	Research has shown chiropractic to be an effective form of health care for back pain, neck pain, headaches, reflux, bedwetting, ear aches, otitis media, leg pains, headaches, migraine, visual disturbances, dizziness, breathing difficulties, asthma, constipation and dysmenorrheal [sic].
(b) Encourage (directly or indirectly) inappropriate, indiscriminate, unnecessary or excessive use of health services;	42 (12%)	Wellness Care. Once your condition has stabilised you then have a choice of continuing Chiropractic care with a focus on preventing the initial condition returning and new conditions appearing. By having regular check-ups and adjustments we can help you maintain and achieve your ideal level of health. Most patients find that periodic chiropractic check-ups help keep them in tip-top shape. Those who are active, have stressful jobs, or want to be their very best, find that a schedule of preventative visits are helpful in the maintenance of good health.
(c) Mislead, either directly, or by implication, use of emphasis, comparison, contrast or omission	205 (57%)	Given the frequency and variety of misleading claims uncovered, examples are presented in table form. (Table 5: Minor Misleading Claims & Table 6: Major Misleading Claims)
(d) Use testimonials or purported testimonials	80 (22%)	Thanks ‘Chiropractor’, I was dead in bed for 3 days and thanks to you I was back on my feet within few days! Strongly recommend. Example of a visual testimonial:
(e) compare professions without evidence	no breaches found	
(f). Claim or imply that a practitioner provides superior services to those provided by other registered health practitioners	5 (1.4%)	Traditional chiropractic vs NeuroStructural Correction. Traditional chiro aka band-aid care …
(g) exaggerate recovery time;	no breaches found	
(h) Lead Audience to Self-Diagnosis	3 (0.8%)	links to videos encouraging viewers to perform each of Contracted leg length test; cervical range of motion test; carpal tunnel test; and a spinal health test.
(i) Abuse the trust of or exploit a lack of knowledge by the target audience (unconscionable conduct)	no breaches found	
(l) Contain language that could cause undue fear or distress	58 (16%)	A LITTLE SECRET: Don’t let symptoms, or the absence of them, be your guide as to how you are doing. Many cancer patients never have a symptom until the first tumor is detected. By then, for many, it is already too late.
(m) Contain any information or material likely to make a person believe his or her health or wellbeing may suffer from not taking or undertaking the health service	16 (5%)	As a short-term solution to overwhelming physical, chemical or emotional stress, spinal joint dysfunction is a brilliant coping strategy. Yet, when this stress response doesn’t resolve in a timely manner, or the stress is chronic, it may lead to other consequences. Do you have undetected spinal joint dysfunctions? Find out!
(n) misrepresent price information	no breaches found	
(o) Unfounded Claims: a practitioner has an exclusive or unique skill or remedy, or that a product is ‘exclusive’ or contains a ‘secret ingredient’	9 (3%)	Integrative Diagnosis is the only complete system for the diagnosis and conservative treatment of muscle, nerve and joint problems … [unlike other chiropractors] we can accurately and quickly diagnose what is causing your lower back pain.
(o)4. Claim or imply that results are always effective	1 (0.3%)	“every adjustment has a positive effect on the brain”
(j, k & p) Combined: Failure to disclose risks, warn of material risks, omit warning statements	32 (9%)	How safe is chiropractic: If the vertebrae are misaligned, the nerves will lack the ability to carry messages that in turn can affect how well our body functions. This can cause problems in the digestive system, anxiety, uneasiness, depression, headaches or ear infections to name a few. A clear example is of an ear infection which can be caused by a bone that is out of alignment. Commonly, migraines, neck pain, back pain or foot pain can be treated or prevented by chiropractic care. Studies have also proved that it can improve blood pressure in patients who have hypertension.
(q) provide a patient or client with an unsolicited appointment time not requested by the patient or client	no breaches	
(r) promote tobacco products, smoking, alcohol, or any other addictive substances or products known to affect health adversely	no breaches	
(s) be vulgar, sensational, contrary to accepted standards of propriety or likely to bring a health profession into disrepute, for example, because the advertising is sexist.	no breaches	

Examples are direct quotes from practitioner advertisements

**Table 4:** All Breaches by Physiotherapists (Number and %) with Examples

Section 5 of the 2014 AG (§5) Category	Physiotherapists with Breaches: Number & (%)	Example
(a) Create unwarranted and unrealistic expectations about service effectiveness	2 (0.53%)	Your physiotherapist will use a combination of joint mobilization, stretching, manual therapy, electrotherapy, ultrasound and structured exercise programs to get you back to 100% health.
(b) Encourage (directly or indirectly) inappropriate, indiscriminate, unnecessary or excessive use of health services;	8 (2%)	Book your Free Initial Assessment today [without terms or conditions]
(c) Mislead, either directly, or by implication, use of emphasis, comparison, contrast or omission	78 (20%)	Given the frequency and variety of misleading claims uncovered, examples are presented in table form. (Table 5: Minor Misleading Claims & Table 6: Major Misleading Claims)
(d) Use testimonials or purported testimonials	179 (47%)	I started going to ‘Suburb’ Physio last year after getting some terrible neck pain from a combination of bad sitting posture at work, and a heavy training schedule. Bob and Jane have done an amazing job at relieving my neck pain! Bob gave me a comprehensive assessment and really took the time to understand what was causing my pain. He gave me exercises to help strengthen the affected muscles and to prevent further injury.
(e) compare professions without evidence	no breaches found	
(f). Claim or imply that a practitioner provides superior services to those provided by other registered health practitioners	51 (11%)	‘Y’ Physiotherapy is Australia’s leading physiotherapy clinic for swimmers.
(g) exaggerate recovery time;	no breaches found	
(h) Lead Audience to Self-Diagnosis	no breaches found	
(i) Abuse the trust of or exploit a lack of knowledge by the target audience (unconscionable conduct)	no breaches found	
(l) Contain language that could cause undue fear or distress	no breaches found	
(m) Contain any information or material likely to make a person believe his or her health or wellbeing may suffer from not taking or undertaking the health service	no breaches found	
(n) misrepresent price information	no breaches found	
(o) Unfounded Claims: a practitioner has an exclusive or unique skill or remedy, or that a product is ‘exclusive’ or contains a ‘secret ingredient’	no breaches found	
(o)4. Claim or imply that results are always effective	8 (2%)	we will find out what the problem is and treat to fix it. We can help you, no matter what your goal is
(j, k & p) Combined: Failure to disclose risks, warn of material risks, omit warning statements	no breaches found	
(q) provide a patient or client with an unsolicited appointment time not requested by the patient or client	no breaches	
(r) promote tobacco products, smoking, alcohol, or any other addictive substances or products known to affect health adversely	no breaches	
(s) be vulgar, sensational, contrary to accepted standards of propriety or likely to bring a health profession into disrepute, for example, because the advertising is sexist.	no breaches	

Examples are direct quotes from practitioner advertisements

**Table 5:** Examples of Minor Misleading Claims as They Appeared on Practitioner’s Websites^a^

Misleading Claim Category	Chiropractors	Physiotherapists
Misrepresenting awards	‘X’ Chiropractic: The Award Winning Spine Experts.^b^	No examples found.
Misrepresenting Qualification	John Chiropractor DC.^c^	No examples found.
Association membership presented as postnominals	Mary Chiropractor BSc, DC, MCAA.^d^	Bob Physiotherapist B AppSc (Physio) MAPA.^e^
Specialization claim	• a specialist chiropractor for more than a decade.	• specialising in the diagnosis and treatment of musculoskeletal dysfunction and sports injuries
Use of the title Dr. without professional clarification	67 of breaches found. No example required. See explanation below.^f^	No examples found.

Explanatory Notes:

^a^These are quotes from practitioners’ webpages

^b^This was a small business award unrelated to spinal expertise

^c^John Chiropractor did not graduate with a Doctor of Chiropractic, rather he graduated with a double degree (Bachelor/Master or double Bachelor) in chiropractic

^d^The letters MCAA mean: Member Chiropractors Association of Australia

^e^The letters MAPA mean: Member Australian Physiotherapy Association. Membership also appears as APAM

^f^If practitioners choose to adopt the title ‘Dr’ in their advertising, and they are not registered medical practitioners, then (whether or not they hold a Doctorate degree or PhD) they should make it clear that they do not hold registration as medical practitioners [26].

Eg. Dr. Walter Lin (Chiropractor)

**Table 6:** Examples of Major Misleading Claims as They Appeared on Chiropractors’ Websites^a,b^

Misleading Claim Category	Chiropractors
Failure to mention adverse outcomes	1. There is ample evidence that chiropractic care is safe for children and NOT A SHRED of evidence that it is harmful or dangerous.
Misuse of the literature	1. In relation to the treatment of neck and back pain, studies have shown that a course of chiropractic care was 250 times safer than a course of anti-inflammatory drugs.^c^ 2. Studies show that mothers under chiropractic care, delivering the first baby, have 25% reduced labour time in comparison to women without care and even 31% shorter labour time in case of pregnancy after the first child.^d^ 3. An Australian study indicates that women consulting with chiropractors during pregnancy are less likely to require a caesarean section after onset of labour or to have a premature birth.^e^ 4. chiropractic care may help with: asthma & allergies, reflux & colic, blood pressure & more. ^f^
Webster technique or claims to affect positioning of an unborn foetus.	1. However, a realignment method, known as the Webster Technique, has a 92% success rate in optimal foetal positioning.
Making unsubstantiated claims.	1. If I had cancer or any illness, I’d rather remove my subluxations, so my nervous system is functioning at 100%. It would be many times worse if I had cancer and a nervous system that isn’t working well.

Explanatory Notes:

^a^There were no Major Misleading Claims by physiotherapists

^b^These are direct quotes from chiropractors’ webpages

^c^This is a commonly seen overreach referenced to Dabbs et al. [52]. Dabbs et al. state “NSAIDs are the most common conventional first-line treatment for most musculoskeletal neck pain”. Dabbs et al. inappropriately reference this to Dillin’s 1992 [53] paper which focuses on the scientific design and concepts of drug management of cervical disk disorders in which steroids, nonsteroidal anti-inflammatory medicines, narcotics, antidepressants and muscle relaxants were discussed. Nowhere in the Dillin paper does it state that NSAIDS are the most common conventional first line treatment for most musculoskeletal neck pain. Dabbs et al. confirm they were unable to find an estimate of the number of patients who are treated with NSAIDs specifically for neck pain of musculoskeletal origin but somehow conclude “This review of the literature found that NSAID treatment for neck pain has a significantly greater risk of serious complications or a death than the use of cervical manipulation”. The number 250 cited by many chiropractors never appears in the Dabbs et al. paper

^d^This is a common claim by chiropractors. The figures are referenced to one poorly conducted, uncontrolled and un-replicated study by J. Fallon reported in 2 publications in 1990 and 1991 [54, 55]

^e^This is an example of selective reporting. This is referenced to a paper highlighting the incidence of adverse birth outcomes and alternative medicine use by Steel et al. [56]. Although the chiropractor’s claim is accurate, important information was omitted. Steel et al. also noted: women under chiropractic care during pregnancy are more likely to experience emotional distress and are also more likely to have an instrumental childbirth

^f^This is an example of claims supported by out of date research. This claim is referenced to the Winsor Autopsies, published in 1921 [57]

### Association membership and AG compliance

There are 2 voluntary professional chiropractic associations within Australia: The Australian Chiropractors Association and Chiropractic Australia. At the time of the audit, the Australian Chiropractors Association (CAA) and Chiropractic Australia (CA) advised that 62% of the chiropractic population were members of a professional association nationally [CAA & CA personal communication 10 August 2018]. Overall, 55.0% (198) of the chiropractic dataset were members of a chiropractic professional association. Amongst these, 72.3% (142) had a breach, whereas 70.7% (114) of non-members had a breach. A chi-square test was performed using Microsoft Excel® to determine whether there was any difference in numbers of breaches between association members and non-members. The chi-square statistic is 0.036 and the *p* value is .85. At *p* < .05 this indicates no significant difference between numbers of breaches by members versus those by non-members. Association membership did not influence the advertising compliance of registered chiropractors.

## Discussion

These data demonstrate that within the samples audited, neither profession exhibited a high level of compliance with the advertising guidelines. As a group, chiropractors had more at-risk advertising and the nature of the at-risk advertising had a greater potential to cause harm. Membership of a professional chiropractic association did not appear to increase compliance by chiropractors. No physiotherapist had at-risk advertising classified as a major misleading breach. Physiotherapist breaches were confined to minor misleading breaches: displaying association membership as postnominals and using testimonials.

Previous research has examined conduct breaches by these professions and claims made in chiropractic patient brochures and on chiropractic college web sites. Within Australia, research in 2018 indicated that both professions had conduct breaches with chiropractors having 6 times more complaints than physiotherapists [16]. Ryan, Too and Bismark found that only a small percentage of the professions’ members are the subject of conduct complaints [16]. Bismark et al. analysed 43,256 complaints against Australian registered health care providers to determine who typically makes complaints. They found 67% of complaints were made by patients or relatives. The rest were made by fellow practitioners (11.9%), employers (10.1%), subjects themselves (5.4%), and other agencies (6.7%) [17].

Elsewhere, at the turn of the twenty-first century, patient brochures from the largest State, Provincial, and National Chiropractic Associations and Research Agencies in Canada and the USA were found to contain many unsubstantiated, potentially harmful claims [28, 29]. More recently, researchers examined World Wide Web claims by chiropractors, amongst others, in Australian, New Zealand, Canada and the United Kingdom and found unsubstantiated, potentially harmful claims to be abundant [30, 31]. These studies provide a clear picture about the volume and distribution of unsubstantiated claims, conduct and advertising complaints and the instigators of complaints.

AHPRA’s annual reports only provide the number of advertising complaints broken down by profession. The rate of advertising compliance and the types of at-risk advertising within the professions is unknown. This appears to be the first study to examine the extent and the nature of practitioner advertising breaches in a representative sample of Australian chiropractors and physiotherapists using advertising guidelines as a standard.

The chiropractic findings are of major concern for two reasons, the first being public safety. Society expects and accepts that professionals advertise their services to assist consumers in making informed choices. To meet societal expectations and legal obligations, advertising must be socially responsible, truthful, appropriate and not misleading or deceptive. Advertising that fails to meet these expectations has the potential to harm. To assist practitioners in fulfilling their obligations, regulators formulated specific rules about advertising of health services to protect the rights of consumers however the data indicate that both professions and chiropractors in particular are not fulfilling their obligations.

The second reason is the high percentage of chiropractors advertising in an unacceptable manner. This raises questions about the profession’s culture and understanding of its obligations under the social contract. It is beyond the scope of this paper to examine this; however, this topic has been the subject of papers by observers both within the profession and external to it over several decades [32–41]. The consensus is, although the profession has many of the trappings of a mainstream healthcare provider, (legislative recognition, high utilization rates, growing global footprint etc.), it is lacking in other key areas such as civic professionalism and upholding the social contract, both of which are critical components within health care [42, 43]. This research reinforces that position.

In this electronic age most health care providers have a web presence and increasingly use social media in their practices in response to consumer demand [44–46]. With the rising use of electronic communication comes increased risk of misleading and deceptive advertising by practitioners. The principal role of health practitioner regulatory authorities is to protect the public from harm.

Traditionally regulatory authorities have been reactive to complaints; however, there is an argument to be made for increased public protection by the authorities becoming proactive. Recent experience by the College of Chiropractors of British Columbia (CCBC) demonstrates that auditing practice websites and linked Facebook pages is a simple, comprehensive and cost-effective way of identifying breaches and achieving compliance with its Efficacy Claims Policy [advertising policy]. The CCBC’s 1200 registrants had 1 months’ notice of an upcoming audit of webpages and social media. Within 2 weeks of commencement of the audit procedure, 97% of the practitioners notified of a potential breach had voluntarily complied with the CCBC’s directive [47]. The CCBC recognizes its audit does not capture all registrant advertising because only just over 70% of registrants have a web presence and only 50% have associated social media pages. However, those registrants who rely on printed material have generated no recent advertising complaints and therefore do not appear to pose the same risk to the public. The CCBC expects that the web page audit will also have a positive effect on non-web-present practitioner behaviour. [E-mail to CCBC (info@chirobc.com) November 21, 2018].

It is conceivable a greater understanding of advertising practices by each profession will encourage practitioners to comply and provide regulators, educators, professional associations with an in-depth understanding of the number and nature of breaches. Perhaps the CBA’s and AHPRA’s enhanced Advertising compliance and enforcement strategy for the National Scheme [11, 48] will have a positive effect on compliance.

### Study strengths and limitations

A key strength of this study is its comprehensiveness. Using representative samples, advertising by 739 practitioners was audited on 214 websites plus associated Facebook pages. Staff profiles, frequently asked question sections, video presentations, and educational materials were scrutinised. When scientific publications were referenced within the advertising they were sourced and reviewed for accuracy. The audit was conducted using a detailed template based on 33 categories of inappropriate advertising. The template was prepared using examples provided within registration board advertising guidelines.

### The study had limitations

Confirmation bias is frequently a problem with this type of research. Both auditors are registered chiropractors with careers involving chiropractic guideline compliance matters and consumer law. This could have made them more sensitive to breaches by chiropractors and, due to their comparative lack of experience with physiotherapist compliance matters, less sensitive to compliance breaches by physiotherapists. While the auditing may have been strengthened by having a physiotherapist conduct the physiotherapy audit, both professions work in musculoskeletal medicine and have the same advertising guidelines. Both auditors are conversant with the musculoskeletal medicine literature and are confident that the physiotherapist audit data are accurate.

Comparability of individual auditor’s findings could be a limitation. This was minimized because each auditor performed ½ the audits in each profession’s sample using a comprehensive breach template developed from the AGs and associated explanatory notes/examples. While there was no formal check of the reliability of the auditors’ findings, an informal check emerged when the data was being inputted into NVivo. Due to the number of group practices, there were about 10 instances in which both auditors had examined the same website for a different group member. In all instances the breach allocation was the same.

Clustering of practitioners due to group practices may skew the results. Locating webpages for 380 physiotherapists required 72 practice websites versus 141 for 359 chiropractors which indicates that the number of physiotherapists per group practice is larger than the number of chiropractors per group practice. While a larger sample from each profession would reduce the possibility of skewed results due to group practice clustering, this limitation can largely be discounted because of the distinction between the physiotherapy results and chiropractic results as depicted in Figs. 1 and 2.

An additional limitation is underreporting of breaches amongst chiropractors. It was not uncommon to find multiple breaches within a single category on a practitioner’s website but only one example was recorded. Similarly, many breaches could be indexed into multiple categories however only the category with the greatest harm potential was used. This was in keeping with the aim of the research, namely to determine what percentage of each profession had websites containing AG breaches with examples included for explanatory purposes.

## Conclusions

Advertising by health care professionals is an accepted part of practice. It informs the public about the profession and the professional enabling the public to make better health care choices. Advertising by the 15 Australian registered health care professions is regulated by a registration board and AHPRA governed by specific advertising guidelines and the National Law. The main objective of this study was to examine advertising by chiropractors and physiotherapists to determine the frequency and nature of advertising guideline breaches. While both professions had advertising that breached the guidelines, breaches by chiropractors were more frequent and more serious.

The study highlights areas for future research. Given that chiropractors are over-represented in both conduct and advertising breaches is there a nexus between the two? Are chiropractors who breach advertising guidelines more likely to generate conduct breaches? Are there alternate compliance measures that would be more effective for chiropractors? Is there something in the professional development of chiropractors and physiotherapists that makes them prone to breaching advertising guidelines? AHPRA, professional associations and educational bodies may find the data from this audit helpful in designing further research and developing interventions that raise compliance by chiropractors and physiotherapists and protect the public from harm.

## Footnotes

1 Where references were used on the website to substantiate claims, these were sourced, and the accuracy of the claims assessed.

2 Both the National Australian Physiotherapy Association and Western Australian APA State Branch were contacted by email and telephone requesting assistance by JKS. Neither would assist on member privacy grounds.
